# The Relationship between Dietary, Serum and Hair Levels of Minerals (Fe, Zn, Cu) and Glucose Metabolism Indices in Obese Type 2 Diabetic Patients

**DOI:** 10.1007/s12011-018-1470-3

**Published:** 2018-08-08

**Authors:** Ewelina Król, Paweł Bogdański, Joanna Suliburska, Zbigniew Krejpcio

**Affiliations:** 1 Institute of Human Nutrition and Dietetics, Poznan University of Life Sciences, 31 Wojska Polskiego, 60-624 Poznan, Poland; 2 Department of Internal Medicine, Metabolic Disorders and Hypertension, Poznan University of Medical Sciences, 84 Szamarzewskiego, 60-569 Poznan, Poland

**Keywords:** Iron, Zinc, Copper, Serum, Hair, Dietary intake, Diabetes type 2 patients

## Abstract

The aim of this study was to assess the levels of Zn, Fe and Cu in the serum and hair, and dietary intake of type 2 diabetic patients and their association with glucose and lipid indices. The study was conducted on 62 people aged 40–78 years (31 diabetic patients and 31 healthy subjects, who were the control group). The content of trace elements in the hair and serum was analysed with the AAS method. The serum insulin, HbA1c, glucose, total cholesterol and triacylglycerol concentrations were measured by means of RIA, HPLC and colorimetric methods, respectively. The diabetic patients were found to have significantly higher dietary iron intake, higher hair Fe and lower serum Zn concentrations than the non-diabetic subjects, while the hair Zn and Cu contents were comparable in both groups. The serum Zn and Cu levels of the diabetic subjects were negatively correlated with the serum glucose, the serum Zn and Cu/Zn ratio was inversely correlated with the serum total cholesterol and the serum insulin level was positively associated with the hair Cu/Zn ratio. The results of this study indicate that the trace element status (Zn, Fe, Cu), as reflected in the blood serum and hair, may be disturbed due to metabolic derangement occurring in diabetes.

## Introduction

According to the WHO estimates, in 2014, there were about 422 million people suffering from diabetes worldwide [1], and in 2030, diabetes will be the seventh leading cause of death [2]. For more than a dozen years, there has been growing interest in the role of minerals in the development, progression and complications in diabetes. There is no doubt that essential minerals play an important role in metabolism. Disturbance in metabolic processes occurring in diabetes mellitus, which is characterised by chronic hyperglycaemia and defects in insulin secretion as well as insulin action, or both, can affect the mineral status in various ways. Recently, Eshak et al. [3] conducted a prospective cohort study in Japan assessing the relationship between dietary intakes of iron, copper and zinc and the risk of type 2 diabetes mellitus (T2DM). The research showed a positive association between dietary total and non-heme iron and copper intake and the risk of T2DM. This relationship was found to be stronger when other factors, such as overweight, old age, smoking and family history of diabetes, overlapped. By contrast, dietary zinc intake was inversely associated with the risk of T2DM. Bao et al. [4] observed that greater dietary intakes of total iron, dietary heme iron as well as supplemental iron were related to higher risk of T2DM in women with gestational diabetes.

Meat intake has been consistently shown to be positively associated with the incidence of type 2 diabetes. Shuang et al. [5] published the results of a systematic review and meta-analysis of cohort studies on protein consumption and T2DM risk. They concluded that red meat and processed meat were risk factors of T2DM, while soy, dairy and dairy products were the protective factors against T2DM. Egg and fish intake does not reduce the risk of T2DM. According to the authors, the results indicate what type of dietary protein and food sources of protein should be considered in the prevention of diabetes [5].

Most recently, Søgaard et al. [6] assessed the association between Fe and the risk of type 1 diabetes (T1DM) based on the results of a systematic literature search. From a total of 931 studies screened, the authors included 4 observational studies evaluating the Fe intake from drinking water or food during early life and the risk of T1DM. One out of the four studies found estimates of the dietary Fe intake to be associated with the risk of T1DM, whereas three studies found no such relationship for estimates of Fe in drinking water. They concluded that the limited number of studies found dietary Fe, but not Fe in drinking water, to be associated with the risk of T1DM [6].

Recently, the HUNT3 study investigated the relationship between 25 elements and type 2 diabetes [7, 8]. The authors concluded that elements such as bromine, cadmium, chromium, iron, nickel, silver and zinc may have played a significant role in the development of diabetes in newly diagnosed type 2 diabetic patients [7]. In another study by this team [8], the prevalence of diabetes was positively correlated with the content of some elements (e.g. boron, calcium and silver) in whole blood but there were no correlations with zinc and copper [8]. It is noteworthy that all these correlations referred to the concentrations of elements in the whole blood, which did not reflect the mineral body status.

Zinc and copper are important minerals in the antioxidant defence system, as they are important cofactors of enzymes, i.e. superoxide dismutase (SOD). Moreover, zinc is necessary for insulin production and storage [9]. Zn can induce an increase in glucose transport into cells as well as potentiate insulin-induced glucose transport [10]. Zinc transporters also play a significant role in the pathogenesis of diabetes [11, 12]. Metabolic changes caused by dysregulation of glucose and lipid metabolism, which is characteristic of diabetes, bring about derangement of the homeostasis of some mineral elements. Both mineral deficiency and excess have been reported in diabetic patients and in animal models of type 2 diabetes. For example, higher amounts of zinc and magnesium are excreted with urine [13, 14], while iron accumulates in the body due to elevated ferritin levels in diabetes [15, 16]. In type 1 diabetes, the elevated copper concentration and decreased zinc in serum were observed [13, 17]. In this group of diabetic patients, the common is iron deficiency anaemia that is associated with higher concentration of glycated haemoglobin [18].

In the literature, there is no information about hair mineral levels in diabetics. In most studies, only serum or plasma mineral contents were used to analyse relationship between trace elements and diabetes. Thus, the aim of this study was to evaluate the relationships between the serum and hair trace element levels (Fe, Zn and Cu), dietary mineral intakes and blood glucose and lipid parameters in healthy and type 2 diabetic patients.

## Material and Methods

### Subjects

The study was conducted on randomly selected 31 healthy and 31 type 2 diabetic patients. The healthy adult subjects (15 males and 16 females, mean age 63.13 ± 10.01 years; BMI 26.05 ± 3.09 kg/m^2^) were the control group. The type 2 diabetic patients were 15 males and 16 females (mean age 61.03 ± 7.96 years; BMI 34.88 ± 4.30 kg/m^2^). The duration of diabetes in diabetic subject was 6.4 ± 3.4 years. All the patients were recruited from the Metabolic Disorders and Hypertension Clinic in Poznań (Poland). Subjects with type 2 diabetes mellitus, without serious complications, such as retinopathy or nephropathy, were enrolled in the study. The exclusion criteria were vitamin-mineral supplementation in the last 3 months; thyroid hormone, oestrogen, progesterone and diuretic therapy; alcohol and smoking addiction; and hair dyeing. The study fully conformed with the standards of the Declaration of Helsinki. The original study protocol was approved by the Human Subjects Oversight Committee, Poznań University of Medical Sciences, Poland (Approval No. 61/2012).

All the participants were under medical supervision. They were regularly checked by their doctors and received medical treatment (sulfonylurea derivatives, biguanides).

The patients attended the Clinic in the morning, after overnight fasting to give venous blood (10 mL) and scalp hair samples (approximately 0.2–0.5 g). The dietary information was gathered during interview (24-h dietary recall from three consecutive days) [19]. The amount of minerals in daily food ratios was calculated with the Dietetic computer software (Dietetyk 2, IŻŻ, Warsaw, Poland). Additionally, anthropometric measurements (body mass, height, BMI index) were recorded. The participants were weighed in light clothes without shoes. Weight was measured to the nearest 0.1 kg, and height was measured to the nearest 0.1 cm. BMI was calculated by dividing the weight (kg) by height squared (m^2^).

### Analytical Methods

#### Blood Biochemical Parameters

The plasma fasting glucose concentration was measured with the hexokinase method. The plasma lipid profile (total cholesterol and triacylglycerol (TAG) concentrations) was determined by means of standard colorimetric methods, using an Olympus AU560 analyser (Tokyo, Japan) [20–23]. The glycated haemoglobin (Hb_A1C_) concentration was measured by means of high pressure liquid chromatography (HPLC, Variant; Bio-Rad, Hercules, CA, USA) [24]. The plasma insulin concentration was determined using microparticle enzyme immunoassay (IMX, Abbott Laboratories) [25]. The efficacy of glucose utilisation was characterised by insulin resistance indices calculated according to the formula of the homeostasis model (HOMA) [26]: HOMA-IR = (Fasting Glucose[mmol/l] × Fasting Insulin[mIU/l]) / 22.5.

#### Trace Elements in Serum and Hair Samples

Whole blood (5 mL) was drained by venipuncture from the median cubital vein using sterile vacutainer tubes (S-Monovette® 9 mL, Sarstedt, Nümbrecht, Germany). The tubes with samples remained in a standing position for about 20–30 min until the blood clotted; then they were centrifuged (at 20 °C, 1500*g* for 10 min). Then, the blood serum was removed, transferred into sterile metal-free plastic tubes and kept frozen at − 80 °C until analysis. Before analysis, the serum samples were defrosted and diluted with a 0.01% Triton X100 solution (Merck KGaA, Darmstadt, Germany).

Scalp hair samples were cut from six places of the occipital region of the head. They were washed (deionised water, acetone, deionised water) and dried until constant mass at 105 °C, according to the procedure recommended by IAEA [27]. Hair samples (approximately 0.2 g) were subsequently transferred into PTFE digestion vessels, treated with 5 mL of 65% nitric acid (Suprapur, Merck KGaA, Darmstadt, Germany) and digested in an MW oven (Mars-5, CEM Corp., Matthews, NC, USA). After cooling to room temperature, the samples were transferred into volumetric flasks (10 mL) with deionised water, and diluted according to the analytical requirements for a given element. The contents of Fe, Zn and Cu in the serum and mineralised hair samples were determined by means of flame atomic absorption spectrometry (AAS-3 spectrometer with BC, Carl-Zeiss, Jena, Germany). The accuracy of mineral measurements was verified using certified reference material—human serum HN2612 (Randox Laboratories, Crumlin, UK). The recovery values of Fe, Zn and Cu in the serum and hair (expressed as percentage of the mean certified values) were as follows: 99%, 102% and 103% in the serum, and 102%, 99% and 95% in the hair, respectively.

#### Statistical Analyses

All data were presented as mean, standard deviation values. The differences between content of minerals in the hair, serum and dietary intake as well as blood biochemical indices were analysed with the Mann-Whitney test. The relations between variables were checked with the Spearman’s rank correlation coefficient. The significance level was set at *α* = 0.05. All statistical analyses were performed using Statistica 13.0 software (Statsoft Inc., Tulsa, USA).

## Results

Table 1 shows the biochemical characteristics of the control and diabetic groups. There were significant differences in the serum glucose, HbA1c, TAG, total cholesterol concentrations and the HOMA-IR index between the diabetic and healthy subjects. In particular, the diabetic subjects (the whole group) had higher levels of serum fasting glucose (by 98%, *p* < 0.001), HbA1C (by 45%, *p* < 0.001), total cholesterol (by 28%, *p* < 0.05) and TAG (by 46%, *p* < 0.05), as well as the HOMA-IR index (by 116%, *p* < 0.001) than the healthy individuals (the whole group).

**Table 1:** Biochemical indices in blood of control and diabetic groups

Parameter	Control (*n* = 31)			Diabetic (*n* = 31)		
	Total	Women (*n* = 15)	Man (*n* = 16)	Total	Women (*n* = 15)	Man (*n* = 16)
FSG (mmol/l)	4.99 ± 0.80^a^	4.74 ± 0.73	5.24 ± 0.79	9.86 ± 3.47^b^	8.65 ± 1.72	10.91 ± 4.26
Serum insulin (mIU/l)	14.67 ± 4.49	12.45 ± 4.39^A^	16.76 ± 3.37^B^	18.62 ± 10.91	16.42 ± 9.98^A^	20.98 ± 11.73^B^
HOMA-IR	3.25 ± 1.12^a^	2.58 ± 0.84^A^	3.92 ± 0.94^B^	7.02 ± 3.76^b^	5.46 ± 3.32^A^	8.59 ± 3.64^B^
HbA1c (%)	5.44 ± 0.44^a^	5.30 ± 0.38	5.57 ± 0.45	7.98 ± 3.43^b^	7.12 ± 1.43	8.78 ± 4.49
T-CHOL (mmol/l)	4.44 ± 0.91^a^	4.73 ± 0.86	4.15 ± 0.86	5.70 ± 2.09^b^	5.35 ± 1.38	6.01 ± 1.42
TAG (mmol/l)	1.60 ± 0.79^a^	1.54 ± 0.80	1.65 ± 0.77	2.33 ± 1.34^b^	2.18 ± 1.30	2.47 ± 1.42

Data are mean ± SD; *FSG* fasting serum glucose concentration, *HbA1c* glycated haemoglobin, *HOMA-IR* homeostasis model assessment for insulin resistance; *T-CHOL* total cholesterol concentration, *TAG* triacylglycerol concentration; in rows, uppercase letters indicate significant differences between women and men at *p* < 0.05; in rows, different lowercase letters indicate significant difference between diabetic and control group at *p* < 0.05

Moreover, there were some sex-dependent differences in certain blood serum indices in both groups. More specifically, the healthy men had significantly higher serum insulin level (by 34.6%, *p* < 0.05) and the HOMA-IR index (by 52%, *p* < 0.05) than the women. The diabetic male patients had markedly higher serum insulin levels (by 28%, *p* < 0.05) and the HOMA-IR index (by 57%, *p* < 0.05) than the diabetic women.

Table 2 shows the Fe, Zn and Cu contents in the serum and hair as well as dietary intakes of these elements in healthy and diabetic subjects. The diabetic subjects (total group) had significantly elevated hair Fe levels (by 100%, *p* < 0.001) and dietary Fe intakes (by 27%, *p* < 0.01) but lower serum Zn levels (by 27%, *p* < 0.05) than the healthy subjects (the whole group). All the other indices were comparable in these groups. There were no sex-dependent differences in the parameters under analysis between the groups.

**Table 2:** The content of minerals in diet, hair and serum

Parameter	Control			Diabetic		
	Total (*n* = 31)	Women (*n* = 15)	Man (*n* = 16)	Total (*n* = 31)	Women (*n* = 15)	Man (*n* = 16)
Fe serum (mg/L)	1.31 ± 0.48	1.34 ± 0.42	1.27 ± 0.53	1.17 ± 0.41	1.09 ± 0.46	1.25 ± 0.37
Hair (μg/g)	11.18 ± 3.69^a^	10.33 ± 3.28	11.99 ± 3.87	22.47 ± 10.21^b^	24.28 ± 9.39	21.88 ± 10.68
Diet (mg/day)	8.64 ± 2.89^a^	7.29 ± 2.24	9.91 ± 2.86	10.99 ± 2.19^b^	10.91 ± 2.78	11.06 ± 1.70
Zn serum (mg/L)	0.80 ± 0.17^a^	0.80 ± 0.20	0.80 ± 0.13	0.59 ± 0.15^b^	0.63 ± 0.07	0.56 ± 0.10
Hair (μg/g)	121.65 ± 33.40	118.38 ± 27.47	124.72 ± 38.14	131.07 ± 63.26	121 ± 52.56	144.42 ± 76.57
Diet (mg/day)	10.36 ± 3.41	8.79 ± 2.70	11.84 ± 3.33	9.90 ± 2.09	9.11 ± 1.81	10.54 ± 2.16
Cu serum (mg/L)	1.04 ± 0.19	1.03 ± 0.18	1.05 ± 0.21	1.07 ± 0.29	1.15 ± 0.29	1.00 ± 0.27
Hair (μg/g)	18.10 ± 9.71	17.91 ± 7.98	18.28 ± 11.18	13.97 ± 6.30	14.72 ± 8.09	13.04 ± 3.31
Diet (mg/day)	1.12 ± 0.41	0.94 ± 0.35	1.30 ± 0.38	1.21 ± 0.31	1.21 ± 0.44	1.18 ± 0.15
Cu/Zn ratio						
Serum	1.37 ± 0.40	1.39 ± 0.48	1.35 ± 0.31	1.51 ± 0.61	1.54 ± 0.64	1.47 ± 0.61
Hair	0.16 ± 0.08	0.16 ± 0.07	0.16 ± 0.10	0.15 ± 0.11	0.16 ± 0.12	0.14 ± 0.10
Diet	0.11 ± 0.03	0.11 ± 0.02	0.12 ± 0.04	0.12 ± 0.02	0.13 ± 0.03	0.11 ± 0.02

Data are mean ± SD; in rows, different lowercase letters indicate significant difference between diabetic and control group at *p* < 0.05

In diabetics with total cholesterol concentration > 4.9 mmol/l, the serum Cu/Zn ratio (*p* < 0.001) and Cu intake (*p* < 0.05) were higher than in those with normalised lipid parameters (Table 3). T2DM patients with TAG > 1.7 mmol/l were characterised by lower serum zinc level (*p* < 0.05).

**Table 3:** The contents of minerals in diet, hair and serum according to glycated haemoglobin, total cholesterol and triacylglycerol concentrations

	HbA1c*		T-CHOL		TAG*	
	< 7% (*n* = 16)	> 7% (*n* = 15)	< 4.9 mmol/l (*n* = 13)	≥ 4.9 mmol/l (*n* = 18)	< 1.7 mmol/l (*n* = 15)	≥ 1.7 mmol/l (*n* = 16)
Fe serum (mg/L)	1.11 ± 0.46	1.25 ± 0.47	1.12 ± 0.45	1.44 ± 0.14	1.09 ± 0.57	1.20 ± 0.30
Hair (μg/g)	19.55 ± 8.79	24.50 ± 12.25	21.80 ± 9.12	24.55 ± 9.87	25.02 ± 16.56	21.22 ± 3.40
Diet (mg/day)	12.01 ± 2.22^a^	10.31 ± 2.02^b^	9.32 ± 1.44	11.43 ± 2.17	10.63 ± 2.79	11.27 ± 2.02
Zn serum (mg/L)	0.57 ± 0.15	0.61 ± 0.17	0.61 ± 0.14	0.56 ± 0.10	0.68 ± 0.10^a^	0.54 ± 0.15^b^
Hair (μg/g)	135.43 ± 57.89	130.56 ± 67.51	134.78 ± 67.56	130.42 ± 58.67	134 ± 45.22	131.97 ± 80.23
Diet (mg/day)	9.61 ± 2.71	10.40 ± 1.54	9.50 ± 2.62	10.06 ± 2.10	8.56 ± 1.52	10.47 ± 2.11
Cu serum (mg/L)	1.09 ± 0.31	1.07 ± 0.29	1.08 ± 0.23	1.06 ± 0.24	1.12 ± 0.29	1.04 ± 0.31
Hair (μg/g)	13.67 ± 4.97	12.13 ± 2.18	16.08 ± 5.54	12.42 ± 3.35	14.98 ± 8.35	12.83 ± 3.40
Diet (mg/day)	1.31 ± 0.38	1.13 ± 0.22	0.92 ± 0.18^a^	1.28 ± 0.29^b^	1.11 ± 0.28	1.26 ± 0.32
Cu/Zn ratio						
Serum	1.54 ± 0.64	1.47 ± 0.61	1.09 ± 0.25^a^	1.78 ± 0.66^b^	1.35 ± 0.58	1.60 ± 0.66
Hair	0.16 ± 0.12	0.14 ± 0.10	0.15 ± 0.09	0.15 ± 0.11	0.15 ± 0.09	0.15 ± 0.13
Diet	0.13 ± 0.01	0.12 ± 0.02	0.11 ± 0.01	0.13 ± 0.02	0.12 ± 0.02	0.12 ± 0.02

Data are mean ± SD; *HbA1c* glycated haemoglobin, *T-CHOL* total cholesterol concentration, *TAG* triacylglycerol concentration; in rows, different lowercase letters indicate significant difference between diabetic and control group at *p* < 0.05

*According to recommendation of Polish Diabetes Association guidelines (2018)

Tables 4, 5, 6, 7 and 8 show the correlation coefficients and *p* values for dietary intake; serum and hair Fe, Zn and Cu levels; the Cu/Zn ratio; and blood biochemical indices.

**Table 4:** Correlation of biochemical indices with serum Fe, Zn and Cu in all control and diabetic subjects

Parameter		Fe serum		Zn serum		Cu serum		Cu/Zn serum	
		*r*	*p*	*r*	*p*	*r*	*p*	*r*	*p*
FSG	Control	0.033	ns	0.124	ns	0.260	ns	− 0.196	ns
	Diabetic	− 0.203	ns	− 0.553	0.002	− 0.376	0.049	0.328	ns
Insulin	Control	− 0.401	0.028	− 0.185	ns	0.295	ns	0.310	ns
	Diabetic	− 0.227	ns	0.470	0.008	0.139	ns	− 0.468	0.008
HbA1c	Control	− 0.133	ns	0.175	ns	− 0.281	ns	− 0.236	ns
	Diabetic	0.305	ns	− 0.138	ns	− 0.105	ns	− 0.011	ns
HOMA-IR	Control	− 0.350	ns	− 0.066	ns	ns	ns	0.141	ns
	Diabetic	− 0.286	ns	0.192	ns	0.004	ns	− 0.276	ns
T-CHOL	Control	0.073	ns	− 0.071	ns	− 0.044	ns	0.005	ns
	Diabetic	0.154	ns	− 0.483	0.007	0.050	ns	0.496	0.005
TAG	Control	0.164	ns	0.241	ns	0.120	ns	− 0.042	ns
	Diabetic	0.175	ns	− 0.318	ns	− 0.090	ns	0.226	ns

*FSG* fasting serum glucose concentration, *HbA1c* glycated haemoglobin, *HOMA-IR* homeostasis model assessment for insulin resistance, *T-CHOL* total cholesterol concentration, *TAG* triacylglycerol concentration, *r* Spearman’s rank correlation coefficient, *p* probability value for correlation, *ns* non-significant

**Table 5:** Correlation of biochemical indices with hair Fe, Zn and Cu in all control and diabetic subjects

Parameter		Fe hair		Zn hair		Cu hair		Cu/Zn hair	
		*r*	*p*	*r*	*p*	*r*	*p*	*r*	*p*
FSG	Control	0.122	ns	0.043	ns	0.283	ns	0.122	ns
	Diabetic	0.204	ns	− 0.280	ns	− 0.114	ns	0.081	ns
Insulin	Control	0.419	0.019	0.311	ns	− 0.101	ns	− 0.040	ns
	Diabetic	− 0.211	ns	− 0.137	ns	0.114	ns	0.449	0.047
HbA1c	Control	− 0.066	ns	0.165	ns	0.332	ns	0.038	ns
	Diabetic	0.181	ns	− 0.146	ns	− 0.070	ns	0.141	ns
HOMA-IR	Control	0.418	0.022	0.291	ns	0.104	ns	0.044	ns
	Diabetic	0.018	ns	− 0.126	ns	0.027	ns	0.231	ns
T-CHOL	Control	0.031	ns	− 0.039	ns	0.003	ns	− 0.079	ns
	Diabetic	0.381	ns	− 0.204	ns	− 0.362	ns	− 0.172	ns
TAG	Control	0.102	ns	− 0.118	ns	− 0.291	0.119	− 0.128	ns
	Diabetic	− 0.155	ns	− 0.300	ns	− 0.308	0.174	0.086	ns

*FSG* fasting serum glucose concentration, *HbA1c* glycated haemoglobin, *HOMA-IR* homeostasis model assessment for insulin resistance, *T-CHOL* total cholesterol concentration, *TAG* triacylglycerol concentration, *r* Spearman’s rank correlation coefficient, *p* probability value for correlation, *ns* non-significant

**Table 6:** Correlation of biochemical indices with dietary Fe, Zn and Cu in all control and diabetic subjects

Parameter		Dietary Fe		Dietary Zn		Dietary Cu		Dietary Cu/Zn	
		*r*	*p*	*r*	*p*	*r*	*p*	*r*	*p*
FSG	Control	0.129	ns	0.099	ns	0.288	ns	0.310	ns
	Diabetic	− 0.119	ns	0.075	ns	0.158	ns	− 0.003	ns
Insulin	Control	0.100	ns	0.116	ns	0.337	ns	0.280	ns
	Diabetic	− 0.105	ns	0.322	ns	− 0.287	ns	− 0.497	0.026
HbA1c	Control	0.222	ns	0.203	ns	0.269	ns	0.315	ns
	Diabetic	− 0.490	0.028	− 0.056	ns	− 0.264	ns	− 0.218	ns
HOMA-IR	Control	0.180	ns	0.175	ns	0.494	0.026	0.363	0.048
	Diabetic	− 0.188	ns	0.298	ns	− 0.125	ns	− 0.415	ns
T-CHOL	Control	− 0.145	ns	− 0.152	ns	− 0.000	ns	− 0.039	ns
	Diabetic	0.046	ns	0.116	ns	0.202	ns	0.128	ns
TAG	Control	− 0.203	ns	− 0.178	ns	− 0.160	ns	0.214	ns
	Diabetic	0.051	ns	0.135	ns	0.090	ns	− 0.065	ns

*FSG* fasting serum glucose concentration, *HbA1c* glycated haemoglobin, *HOMA-IR* homeostasis model assessment for insulin resistance, *T-CHOL* total cholesterol concentration, *TAG* triacylglycerol concentration, *r* Spearman’s rank correlation coefficient, *p* probability value for correlation, *ns* non-significant

**Table 7:** Correlation between minerals in serum, hair and dietary intake in control and diabetic subjects

Parameter		Dietary Fe		Dietary Zn		Dietary Cu		Dietary Cu/Zn	
		*r*	*p*	*r*	*p*	*r*	*p*	*r*	*p*
Fe serum	Control	0.199	ns	0.209	ns	− 0.209	ns	0.047	ns
	Diabetic	0.133	ns	0.245	ns	− 0.119	ns	0.065	ns
Zn serum	Control	− 0.017	ns	0.010	ns	− 0.003	ns	0.097	ns
	Diabetic	− 0.475	0.034	− 0.135	ns	− 0.534	0.015	− 0.338	ns
Cu serum	Control	− 0.054	ns	0.209	ns	0.138	ns	0.024	ns
	Diabetic	− 0.137	ns	− 0.313	ns	− 0.146	ns	0.179	ns
Cu/Zn serum	Control	− 0.065	ns	− 0.083	ns	0.106	ns	− 0.011	ns
	Diabetic	0.379	ns	− 0.218	ns	0.374	ns	− 0.541	0.014
Fe hair	Control	− 0.112	ns	− 0.111	ns	0.220	ns	0.334	ns
	Diabetic	− 0.139	ns	0.097	ns	0.098	ns	0.130	ns
Zn hair	Control	− 0.204	ns	− 0.219	ns	0.120	ns	0.133	ns
	Diabetic	0.029	ns	0.268	ns	0.057	ns	− 0.164	ns
Cu hair	Control	0.007	ns	− 0.009	ns	0.023	ns	− 0.051	ns
	Diabetic	0.125	ns	− 0.061	ns	0.011	ns	0.043	ns
Cu/Zn hair	Control	− 0.207	ns	− 0.191	ns	− 0.101	ns	0.175	ns
	Diabetic	− 0.011	ns	0.005	ns	− 0.038	ns	0.027	ns

*r* Spearman’s rank correlation coefficient, *p* probability value for correlation, *ns* non-significant

**Table 8:** Correlation between minerals in serum and hair in control and diabetic subjects

Parameter		Fe hair		Zn hair		Cu hair		Cu/Zn hair	
		*r*	*p*	*r*	*p*	*r*	*p*	*r*	*p*
Fe serum	Control	− 0.420	0.021	− 0.153	ns	− 0.001	ns	− 0.085	ns
	Diabetic	− 0.809	0.014	0.095	ns	0.048	ns	0.042	ns
Zn serum	Control	0.099	ns	0.109	ns	0.163	ns	0.003	ns
	Diabetic	− 0.208	ns	0.008	ns	0.159	ns	0.439	ns
Cu serum	Control	0.122	ns	0.259	ns	− 0.132	ns	− 0.336	ns
	Diabetic	− 0.330	ns	− 0.110	ns	− 0.223	ns	− 0.043	ns
Cu/Zn serum	Control	0.076	ns	0.076	ns	− 0.169	ns	− 0.226	ns
	Diabetic	− 0.008	ns	− 0.077	ns	− 0.371	ns	− 0.546	0.013

*r* Spearman’s rank correlation coefficient, *p* probability value for correlation, *ns* non-significant

The healthy (control) subjects were characterised by significant (*p* < 0.05) correlations between certain indices, namely, a positive correlation between the serum insulin level and hair Fe content (*r* = 0.419, *p* = 0.019), the HOMA-IR index and hair Fe and Cu contents (*r* = 0.418, *p* = 0.022; *r* = 0.494, *p* = 0.06, respectively). There were negative correlations between the serum insulin and Fe levels (*r* = − 0.401; *p* < 0.05), and the serum Fe and hair Fe levels (*r* = − 0.420, *p* = 0.021).

The diabetic subjects were characterised by different correlations than the control group. In particular, there were negative correlations between the serum insulin and dietary Cu/Zn ratio (*r* = − 0.497, *p* = 0.026). There was negative correlation in the serum Cu/Zn ratio and serum insulin (*r* = − 0.468, p 0.008), between the blood HbA1C concentration and dietary Fe (*r* = − 0.490, *p* = 0.028), the serum Zn and dietary Fe and Cu intake (*r* = − 0.475, *p* = 0.034; *r* = − 0.534, *p* = 0.034, respectively), the serum Fe level and hair Fe content (*r* = − 0.809, *p* = 0.014) and the serum Zn/Cu ratio and hair Zn/Cu ratio (*r* = − 0.546, *p* = 0.013).

## Discussion

The authors of this study evaluated the relationships between the serum, hair and dietary zinc (Zn), copper (Cu) and iron (Fe) levels and selected blood serum biochemical indices in healthy and diabetic subjects. It is known that zinc, copper and iron are essential minerals for a variety of biomolecules to maintain the normal cell structure, function and proliferation. These elements can be toxic in excessive amounts, especially in certain genetic disorders (i.e. hemochromatosis, Wilson’s disease). The homeostasis of Zn, Cu and Fe results from a tightly coordinated regulation by different proteins involved in their uptake, excretion and intracellular storage/trafficking [28]. The appropriate dietary intake of these minerals is necessary to maintain overall physiological functions, including proper glucose and lipid metabolism. Abnormal metabolism of Zn, Cu and Fe can lead to chronic pathogeneses, such as diabetes or diabetic complications. Cu^+2^ and Fe^+2^ under a non-protein-binding condition, through the Fenton reaction, can generate various reactive oxygen species, damaging tissues and cells [28].

Many studies confirmed that the metabolic derangement of glucose and lipid metabolism occurring in diabetes mellitus affects Zn, Cu and Fe levels in body fluids and tissues [29–33], which depend on the severity of glucose intolerance and accompanying complications [34]. Also, abnormal metabolism of Zn, Cu and Fe can further accelerate diabetic complications. Therefore, it is important to understand the mechanisms involved in these processes.

Zinc is a cofactor of plethora of proteins, insulin production and stability and various enzymes engaged in the antioxidative defence systems (i.e. SOD). According to some reports, zinc intake as well as higher dietary zinc/iron ratio can decrease the risk of type 2 diabetes [35, 36]. It is known that the solute carrier family 30 member 8 gene (SLC30A8) encodes a zinc transporter in pancreatic beta cells and that the major C-allele of a missense variant (rs13266634; C/T; R325W) in SLC30A8 is associated with increased risk of type 2 diabetes (T2D). Drake et al. [12] hypothesised that the association between zinc intake and T2DM may be modified by the SLC30A8 genotype. The researchers concluded that zinc supplementation and a high zinc/iron intake ratio may lower the risk of T2DM, but these relations could be modified by obesity and the SLC30A8 genotype.

As many studies report, the zinc status is disturbed in diabetes [16, 32, 33, 37–40]. Zinc deficiency in diabetes could result from disturbed mechanisms of intestinal absorption and urinary excretion. The compensatory mechanisms of Zn homeostasis are inefficient and the urinary zinc excretion is increased, which results in zinc deficiency. The following symptoms of Zn deficiency have been reported in diabetic patients: impaired wound healing, decreased cell-mediated immunity and taste acuity [41]. Therefore, some authors advise dietary zinc supplementation in obesity and prediabetes states [12, 42]. This intervention can compensate for the excessive Zn loss, improve the Zn status, fasting plasma glucose, insulin sensitivity and β-cell function [42].

In our study, the diabetic patients had significantly lower serum Zn concentration (by 27%, *p* < 0.05) than the healthy subjects, despite the comparable dietary Zn intake. It is difficult to assess the Zn status in humans due to the lack of relevant Zn biomarkers, because effective regulation of zinc homeostasis buffers the functional response to dietary deficiency or excess. Usually, assessment of the Zn status is based on analysis of the Zn content in available tissues (i.e. serum/plasma, erythrocyte, lymphocyte, salivary, hair, nail) and certain Zn-dependent biomolecules (i.e. plasma aminolevulinic acid dehydratase, extracellular superoxide dismutase, lymphocyte ecto-5′-nucleotidase, T lymphocyte metallothionein−2A mRNA, carbonic anhydrase, neutrophil alkaline phosphatase, erythrocyte membrane alkaline phosphatase) [43].

The Zn content in the blood serum, plasma or hair does not reflect the Zn body status. The blood (serum) Zn level may represent the current (circulatory) pool of this mineral, which depends on various factors, such as the current dietary intake, intestinal absorption and urinary excretion. Homeostatic mechanisms regulate Zn concentration in the storage and functional pools. Despite various limitations of hair mineral analysis, it is considered an alternative, conditionally useful method of assessment of the mineral status. The hair Zn level is affected by a variety of internal and external factors, i.e. dietary Zn, protein intake, blood Zn concentration, other factors determining the hair growth, environmental factors, etc. Both positive and negative correlations between blood serum/plasma Zn levels have been reported in healthy and diabetic subjects [44, 45].

The role of copper in the development of diabetes and its complications is not entirely clear. On the one hand, Cu is involved in some redox reactions and acts as a pro-oxidant. Cu^+2^ ions can generate reactive oxygen species and lead to oxidative damage of cells through the Fenton reaction, under a non-protein-binding condition [46]. The homeostasis of copper is regulated by ATPases, especially ATP7A, which provides Cu to the enzymes that need this element as a cofactor during synthesis. Most of Cu is transferred to the liver and bound to ceruloplasmin and then by this enzyme to distinct tissues [47]. A recent in vitro study on hepatic cells conducted by that team of researchers showed that at normal Cu concentration, the activity of ATP7B (the enzyme responsible for binding Cu with bile and its excretion) depends on insulin and glucagon concentrations and their ratio. Thus, dysregulation of these hormones, which are associated with obesity and diabetes, could affect the copper status [48]. Another factor that should be taken into account is inflammation. The level of ceruloplasmin rises during inflammation [49] and diabetes [50]. According to Qiu et al. [51], an increased Cu level in diabetes may be caused by higher serum ceruloplasmin levels.

In in vitro, proteomics study, it was found that elevated protein glycation might be associated with Cu deficiency and with excessive Cu(II) concentrations [52]. The data from clinical studies on diabetic patients are contradictory. In some studies, the serum or plasma copper level was increased [31, 34, 37, 44, 46, 53] or the same as in the control group [54].

In Slovakia, Victorinova et al. [46] investigated the association between the glycated haemoglobin level and serum trace element level (Zn, Cu) in healthy and diabetic patients. The authors found increased serum copper and decreased zinc concentrations in the diabetic subjects. There was also a positive correlation between glycated haemoglobin levels and Cu and Cu/Zn ratio, as well as a negative correlation with the serum Zn level, which was more noticeable when HbA1c was higher than 8%.

Xu et al. [13] studied the relationship between the serum Zn and Cu concentration in people with type 1 diabetes, type 2 diabetes, impaired fasting glucose (IFG) or impaired glucose tolerance (IGT). The Cu concentration in the serum of the patients with IFG, IGT and T2DM was higher than in the serum of age/sex-matched control subjects, while the Zn concentration was slightly lower in the type 2 diabetics. Additionally, there was a positive correlation between the serum Cu concentration and HbA1c in the patients with IFG and type 2 diabetics. On the other hand, the type 1 diabetics were characterised by an inverse association between the serum Cu and serum glucose levels. Our study showed an inverse relationship between the serum glucose and serum Cu levels (*r* = − 0.376, *p* < 0.05) in type 2 diabetics. Skalnaya et al. [37] noted similar results in a study on prediabetic and type 2 diabetic women.

Our study also assessed the relations between the Fe, Zn and Cu contents in the serum, hair and diet and biochemical indices. The diabetics were characterised by inverse correlation between the serum total cholesterol and serum Zn levels and positive the serum Cu/Zn ratio. Similarly, Wolide et al. [55] found negative correlations between the serum Zn and Fe levels and the total and LDL, the serum Zn and triacylglycerol levels.

The relationship between T2DM and iron metabolism has gained interest both in research and clinical practice [15, 56]. There was scientific evidence for the influence of elevated serum ferritin levels on IR and T2DM either because of increased body iron stores or due to inflammatory diseases [57–59]. Elevated levels of Fe stores (as ferritin levels) were recognised as a feature of T2DM. However, the relationship between Fe levels and T2DM is complex and it has not been fully investigated. It is known that insulin stimulates ferritin synthesis and activates Fe upload and that Fe influences the insulin inhibition of glucose production from the liver [60]. Hernandez et al. [61] reported that the ferritin concentration in T2DM patients was 2.5 times higher than in healthy people, although the rate of transferrin receptors showed no significant difference. It suggested that increased serum ferritin and negative iron levels may have been caused by an inflammatory problem rather than iron overload. These observations were supported in the study by the Fatima’s researchers, where the authors presumed that increased ferritin concentration may affect glucose homeostasis, leading to insulin resistance and inflammatory changes, even without apparent iron overload [62]. The serum ferritin concentration may be an indicator of systemic fat content and degree of insulin resistance [63].

In our study, the T2DM patients were evidently obese, had significantly higher level of the hair Fe as well as higher dietary Fe intake (although Fe intakes were below the RDI in both groups under study) than the healthy subjects. The serum Fe levels were comparable in both groups, but this biomarker did not reflect the overall Fe status. Judging only by the low dietary Fe intake, both groups might have had some degree of Fe deficiency. However, this statement should be supported by relevant biomarkers (i.e. haemoglobin, TIBC, transferrin receptor, ferritin), which were not analysed. The elevated hair Fe content in the diabetic patients may have been caused by increased inflammatory changes in T2DM or differences in dietary Fe intake (including heme and non-heme Fe). Similarly, Kazi et al. [22] reported that both diabetic women and men had higher hair Fe levels than healthy subjects. It may have been caused by pharmacological treatment which affected the mineral bioavailability and status. Wolide et al. [48] reported that type 2 diabetic patients taking oral hypoglycaemic drugs had higher levels of iron and ferritin in the serum than patients treated with insulin injections. On the other hand, therapy with metformin did not affect serum Zn and Cu level in T2DM patients [64].

Most studies linking the trace elements status with diabetes were based only on limited biomarkers, namely on serum or plasma mineral contents. For many years, the potential usefulness of hair analysis in clinical diagnostics has been discussed without clear conclusions. The content of trace elements in hair depends on many internal and external factors, i.e. age, sex, dietary intake, health status and environmental factors. Another disadvantage of hair mineral analysis as a diagnostic tool is the lack of commonly accepted reference values for elements [65]. Another obstacle to hair analysis is disagreement about setting standardised methodological procedures for sample preparation, processing and determination [65, 66].

Chen et al. [67] discussed the possible usefulness of element analysis for diagnosing diabetes mellitus. The authors compared hair and urine as diagnostic materials in clinical practice and concluded that hair mineral analysis had more advantages than urine analysis due to practical aspects, i.e. the concentration of trace metals in hair was higher than in urine.

In most studies, the levels of minerals in biological samples were analysed; in this study, we also assessed dietary intake of elements. The weakness of this study is relatively small sample size and fact that the inflammatory markers were not assessed. We did not assess the amount of these metals excreted with urine. One of the symptoms of diabetes is polyuria, which can be another potential factor affecting the mineral status. Xu et al. [13] and el-Yazigi et al. [14] reported that diabetic patients had greater total urinary mineral excretion, even though the mean concentration of elements in urine was lower or within a normal range.

## Conclusions

The study showed disturbances in some mineral status indices. The diabetics had lower serum zinc concentrations but higher hair iron levels. The strongest inverse associations between minerals and biochemical indices were found for the serum Zn and glucose and total cholesterol concentrations. The serum Cu level was negatively correlated with the serum glucose level, whereas the serum Zn/Cu ratio was negatively correlated with the total cholesterol concentration. The hair Zn/Cu ratio was inversely correlated with the serum insulin level in diabetic patients. It suggests that diabetic patients should modify their diet, especially in respect to zinc and iron intake. In some cases, zinc supplementation should be considered to improve glucose and lipid indices in T2DM.
